# Laparoscopic-Assisted Vaginal Hysterectomy With Dense Bladder Adhesions and Absent Cervix: A Case Report With a Descriptive Video of the Entire Procedure

**DOI:** 10.7759/cureus.57482

**Published:** 2024-04-02

**Authors:** Jerrelyn J Inocencio-Diaz, Felice J Liang, Boris B Boyanovsky

**Affiliations:** 1 Department of Clinical Sciences, Kaiser Permanente Bernard J. Tyson School of Medicine, Pasadena, USA; 2 Clinical Sciences, Southern California Permanente Medical Group., Pasadena, USA; 3 Department of Biomedical Sciences, Rocky Vista University College of Osteopathic Medicine, Ivins, USA

**Keywords:** post-operative recovery, salpingo-oopherectomy, robotic-assisted laparoscopic gynecologic surgery, multiparous women, minimally invasive gynecologic surgery

## Abstract

Hysterectomy is one of the most frequently performed surgical procedures in the United States. Hysterectomy for benign gynecological reasons can be performed through several approaches: abdominal, laparoscopic, laparoscopically assisted vaginal, robotic-assisted, and vaginal natural orifice hysterectomy. The choice of approach is strongly influenced by factors such as previous procedures, safety, and recovery process. Currently, vaginal hysterectomy, laparoscopic-assisted vaginal hysterectomy (LAVH), assisted vaginal hysterectomy, and robotic-assisted vaginal hysterectomy are considered minimally invasive approaches with multiple benefits to the patient such as less trauma, shorter operative time, and shorter postoperative period. However, in patients with pelvic adhesions, adhesions within the abdominal cavity, especially omental adhesions to the abdominal wall, and adhesions between the uterus and the bladder caused by multiple cesarian sections or prior surgery on the cervix, these minimally invasive approaches are problematic. In this report, we describe in detail our approach to LAVH in a patient with severe abdominal adhesions and an absent cervix. We believe that our approach is safe and relatively fast compared to an open abdominal procedure and, therefore, it may help gynecologic surgeons-in-training nationwide.

## Introduction

Hysterectomy is one of the most frequently performed medical procedures in the United States [1,2]. Many benign conditions, such as fibroids causing abnormal bleeding, pelvic pain, and recurrent cervical dysplasia may be indications to perform hysterectomy [1]. There are several general approaches to hysterectomy for benign gynecological reasons, such as abdominal, laparoscopic, robotic-assisted, and vaginal natural orifice hysterectomy [2,3].

Vaginal hysterectomy, laparoscopic vaginal hysterectomy, and robotic-assisted vaginal hysterectomy are considered minimally invasive approaches which avoid a large abdominal incision, compared to abdominal hysterectomy [1]. However, minimally invasive hysterectomy in patients with adhesions and ill-defined planes of dissection due to previous procedures represents a challenge to surgeons. For example, vaginal hysterectomy leads to a faster return to normal activities [3,4]. However, in patients with dense pelvic adhesions, limited uterine descensus, narrow vaginal opening, and immobile uteri, a vaginal approach is not feasible [5]. At the same time, ideally, minimally invasive hysterectomy should remain the method of choice as it is less intrusive by employing smaller incisions, which results in improved post-operative recovery, less pain, less infection, improved safety, and better wound healing for the patient [1,6].

A considerable disadvantage of laparoscopic hysterectomy is the increased rate of urinary tract injuries, compared to vaginal hysterectomy [6,7]. The laparoscopic-assisted vaginal hysterectomy (LAVH) technique was developed as a hybrid between laparoscopic and vaginal approaches utilizing small incisions in the abdomen, creating pneumoperitoneum, and usage of a camera to visualize and detach the uterus intracorporeally with the aid of specialized instruments. Subsequently, the uterus is removed through the vagina. Most earlier studies and a recent Cochrane review noted an increased rate of ureteral injury with LAVH compared to abdominal hysterectomy [7]. The risk of ureteral injury with LAVH persists, although more recent studies have shown that the risk is decreasing, possibly due to improved surgical technique [7,8].

Lately, robotic-assisted laparoscopic hysterectomy has gained speed; however, it comes at a high price both to purchase the robotic console and also the intra-hospital costs with maintenance and supplies. Isono et al. report that robotic-assisted laparoscopic hysterectomy has sufficient advantages over abdominal [9]. Adhesions within the abdominal cavity, especially omental adhesions to the abdominal wall and adhesions between the uterus and the bladder caused by multiple cesarian sections or prior surgery on the cervix, such as cervical conization, are commonly seen and represent a considerable challenge to surgeons who perform robotic-assisted or laparoscopically assisted hysterectomy and leave abdominal hysterectomy a viable approach despite its obvious insufficiencies. It is intuitive that neither abdominal hysterectomy nor robotic-assisted hysterectomy is the most optimal approach in more complex cases. Therefore, improving the LAVH technique remains the goal to benefit from its advantages [10,11].

In this work, we describe in detail our approach to LAVH. We believe that our approach is safe and relatively fast compared to an open abdominal procedure. Therefore, we believe that it may help gynecologic surgeons-in-training nationwide.

## Case presentation

The patient was a 54-year-old female (G5P5) with a history of three cesarian sections, two loop electrosurgical excision procedure (LEEP), and one cervical cone procedure. There was dense adhesion of the bladder to the lower uterine segment and the absence of a significant portion of the cervix, which resulted in close proximity of the ureter to the uterine vessels. Therefore, the vaginal hysterectomy and bilateral salpingo-oophorectomy represented a challenging surgery. 

Procedure

We are providing step-by-step instructions to perform the surgery, highlighting the important points and challenges at each step. We are also providing figures with crucial moments of the surgery. A narrated video (see Appendices) is available to observe the whole procedure.

Step 1. Laparoscopic Access to the Abdominal Cavity to Explore and Reveal Adhesions

**Figure 1:**
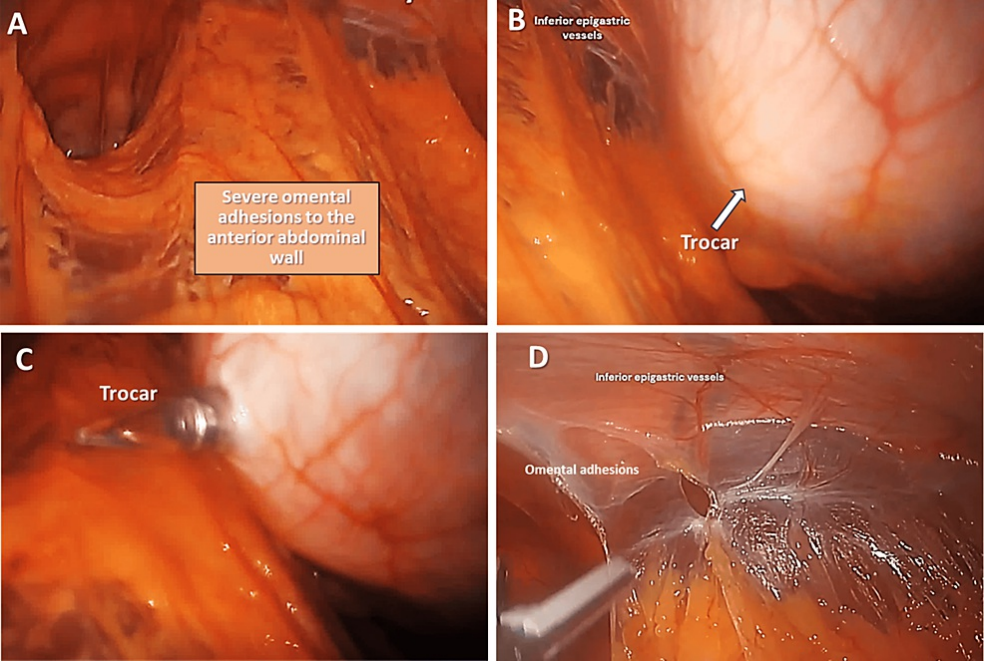
(A) Laparoscopic visualization of omental adhesions completely obscuring the pelvic organs; (B) Visualization of the inferior epigastric vessels; (C) Trocar placement avoiding the inferior epigastric vessels; (D) Cauterization is utilized to detach omental adhesions.

Initially, a trocar placed in the left upper quadrant revealed the lower abdominal cavity with severe omental adhesions, completely obscuring the uterus, fallopian tubes, and ovaries (Figure 1A). Second and third trocars were placed to access the adhesions and help avoid the inferior epigastric vessels. The second trocar was placed in the right upper quadrant, approximately 2 cm lateral to the falciform ligament, which helped to visualize the inferior epigastric vessels (Figure 1B). The third trocar was placed in the right lower quadrant with care to avoid the right inferior epigastric arteries. The fourth trocar was placed in the left lower quadrant with care to avoid the left inferior epigastric vessels (Figure 1C). Severance of the inferior epigastric vessels could result in significant bleeding. Removing the omental adhesions should also be performed cautiously to avoid significant bleeding if the inferior epigastric vessels are damaged (Figure 1D).

Step 2. Exposing the Uterus and Ovaries

**Figure 2:**
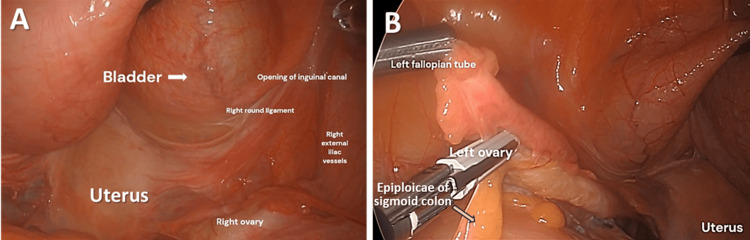
(A) Retracted bladder and visualization of the uterus: adhesions of epiploic appendices to the left fallopian tube and filmy adhesion of colon to left ovary; (B) Cauterization of the round ligament of uterus. Secondary structures are labeled with smaller font.

Clearing the adhesions allows visualization of the pelvic structures. The bladder was retracted and the uterus was exposed (Figure 2A). The right round ligament was seen attaching the uterus to the inguinal canal (Figure 2A). The ovaries are suspended in the pelvis through the suspensory ovarian ligaments (a.k.a. infundibulopelvic ligament (IP)) containing the ovarian vessels. Here, adhesions of the epiploic appendices of the colon to the fallopian tubes and film adhesion of the colon to the left ovary were observed (Figure 2B). Blunt separation of the adhesions from the tubes was performed to expose the ovaries.

Step 3. Exposure and Cauterization of the Round Ligament of the Uterus

**Figure 3:**
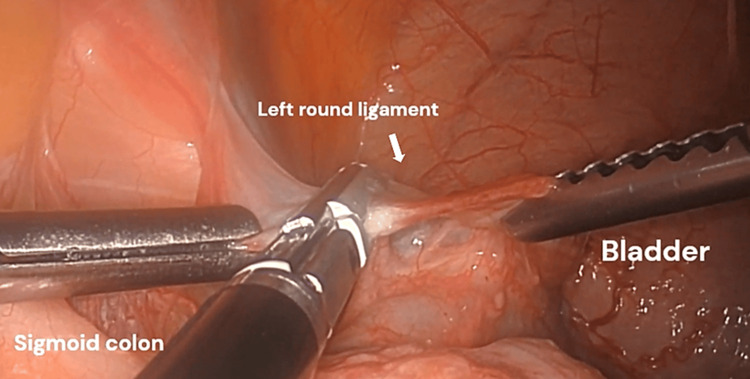
Left round ligament dissection. The bladder is filled and the uterus is not retracted cranially. The cervix is atrophic and so is the uterus, which is why it appears very close to the round ligament.

The left round ligament was cauterized and cut to free the uterus from the left side wall and improve mobility of the uterus superiorly and medially to further increase the distance between the uterine vessels and the ureter and bladder (Figure 3).

Step 4. Partition of the Ureter in Latsko’s Space of the Avascular Pararectal Area

**Figure 4:**
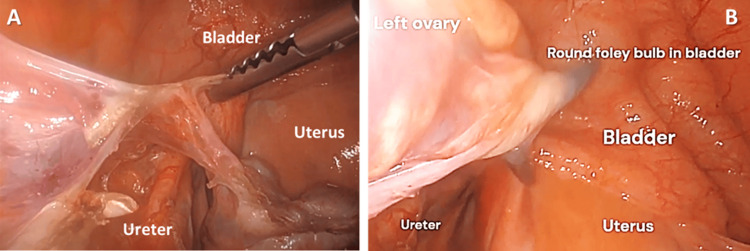
(A) Identification of left ureter in Latsko’s space; (B) Peritoneal window created to separate the infundibulopelvic ligament from the ureter.

First, the ureter was found in the perirectal space (Figure 4A). Then, a peritoneal window was created between the infundibulopelvic (IP) ligament and the ureter to prevent injury to the ureter during the transection of the IP ligament containing the uterine vessels (Figure 4B) [1,2].

Step 5. Cauterization of the IP Ligament After it has been Isolated and the Ureter Retracted

**Figure 5:**
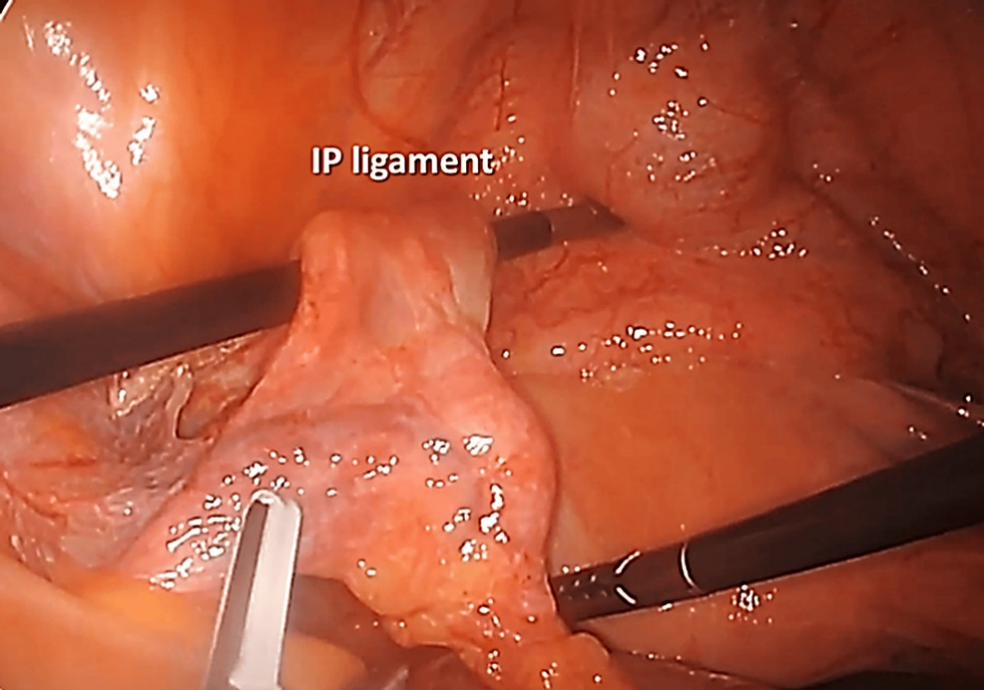
Cauterization of the IP ligament; the ureter is beneath the left black instrument (not visible). IP: infundibulopelvic

Cauterization of the left IP ligament containing the ovarian artery and vein was done after a peritoneal window was created to separate it from the ureter (Figure 5). 

Step 6. Dissection of Anterior and Posterior Peritoneum of the Left Broad Ligament to Identify the Cardinal Ligament Containing the Uterine Vessels

**Figure 6:**
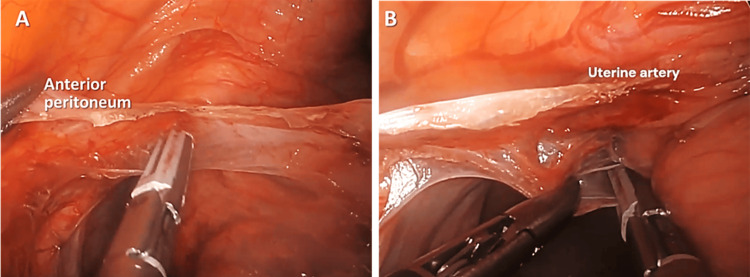
(A) Skeletonization of broad ligament; (B) The posterior peritoneum of the broad ligament is cauterized and cut to allow the ureter to retract further laterally away from the uterine vessels.

Separation of the anterior and posterior peritoneum of the broad ligament to identify the cardinal ligament containing the uterine vessels was performed (Figure 6).

Step 7. Creation of Bladder Flap and Identification of Ureter and Uterine Arteries

**Figure 7:**
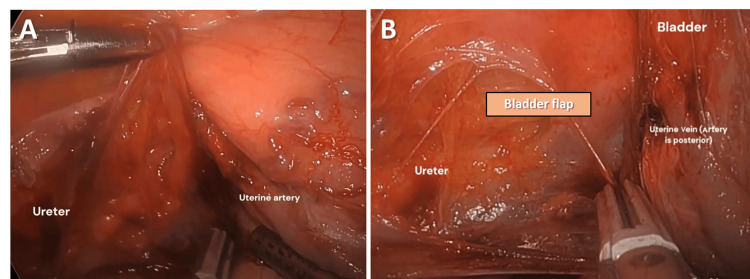
(A) Identification of the ureter; (B) Development of the bladder flap.

Creation of a bladder flap was utilized to allow the bladder and ureter to detach from the uterus and prevent injury. This step involved the dissection of connective tissue between the left uterine artery and vein and of the left ureter (Figure 7A). Creation of a bladder flap further dissected the bladder and ureter away from the uterine artery (Figure 7B). It was important to observe the ureter peristalsis for proper identification. The ureter runs inferiorly to the uterine artery and vein in the cardinal ligament (water under the bridge mnemonic, where “water” is the urine in the ureter). The bladder flap was created by entering the avascular space of the vesicovaginal space. The development of the bladder flap included separation of the loose connective tissues and dense adhesive bands formed from the inflammation-mediated tissue repair at the time of the patient's prior cesarean deliveries. 

Step 8. Cauterization of Cardinal Ligament With Uterine Vessels

**Figure 8:**
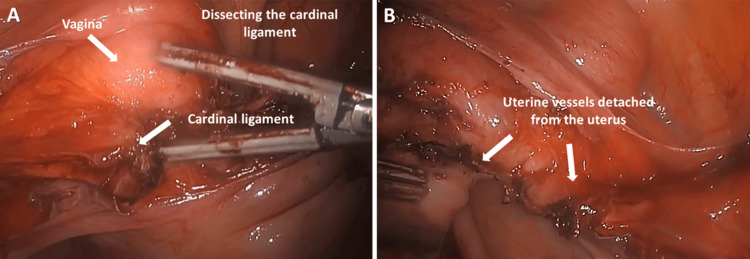
(A) Cauterization of the cardinal ligament containing artery and vein; (B) Cauterized and cut cardinal ligament.

The left cardinal ligament containing the uterine artery and vein was cauterized and cut. Attention was then placed on the right side (Figure 8).

Step 9. Creating the Bladder Flap on the Right Side and Cauterization of the Right Round Ligament.

**Figure 9:**
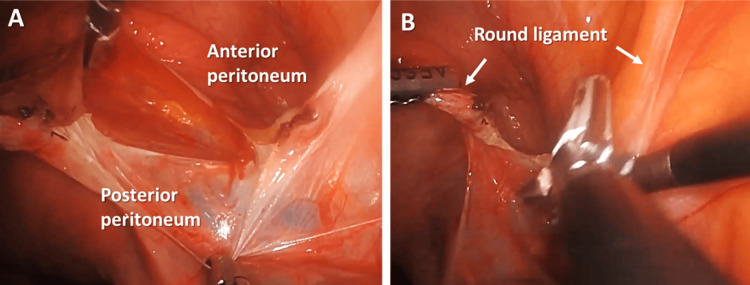
(A) Bladder flap; (B) Right round ligament dissection.

The bladder flap was developed further by cauterizing and cutting the anterior peritoneum towards the previously cut end on the left side. The creation of the bladder flap allowed the bladder and ureter to retract away from the uterus. This decreased the risk of injury to these structures at the time of cauterization of the uterine vessels (Figure 9).

Step 10. Right IP Ligament Cauterization

**Figure 10:**
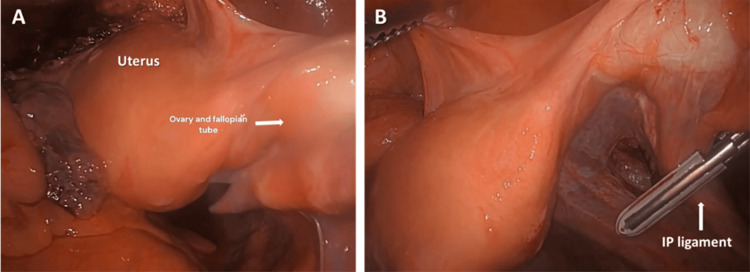
Right IP ligament cauterization. IP: infundibulopelvic

Similarly to the left side, the right ureter was identified, and a peritoneal window was created between the right ureter and the right IP ligament before the IP ligament was cauterized and cut to avoid injury to the ureter (Figure 10).

Step 11. Ureter Preservation During Right Cardinal Ligament Cauterization

**Figure 11:**
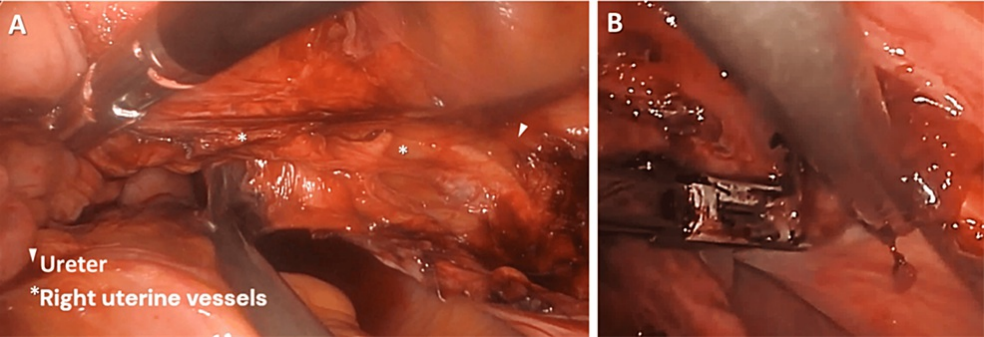
(A) Ureter entering the bladder tunnel under the uterine vessels; (B) Cauterization of right cardinal ligament at the insertion to the uterus and away from the ureter.

The ureter was identified and its entry into the bladder tunnel was dissected, revealing its relation directly under the uterine artery and vein. The right cardinal ligament containing the uterine vessels was cauterized and cut away from the location of the ureter. The bladder and ureter was now fully retracted away from the operative field. The right cardinal ligament was further cauterized and cut while the ureter was further retracted away with a blunt instrument (Figure 11). Again, care was taken to retract the left ureter laterally to avoid injury.

Step 12. Dissection of the Left Uterosacral Ligaments

**Figure 12:**
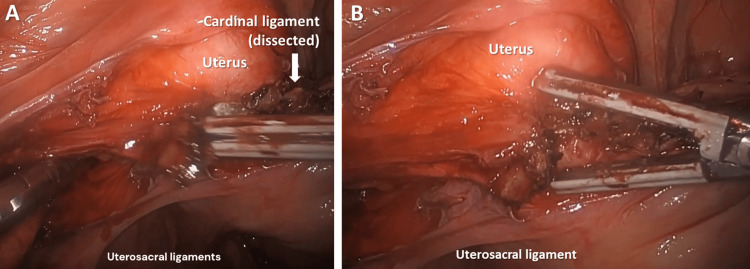
(A) Uterosacral ligaments cauterized and cut; (B) A closer view of the same step.

The same procedure was repeated on the right side, also detaching the right uterosacral ligament from the uterus (Figure 12).

Step 13. Uterine Removal

**Figure 13:**
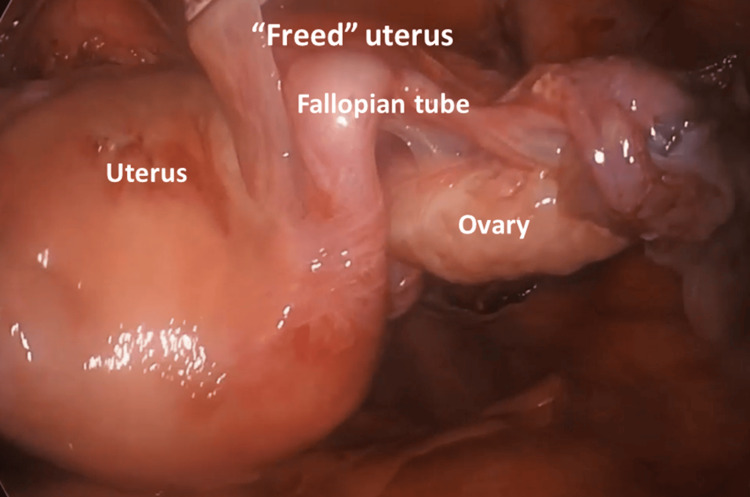
Uterus is “freed” in the abdominal cavity and prepared for extraction through the vagina.

At this point, attention was placed on the vaginal canal. The remnant of the cervix and uterus was detached from the vagina, and the uterus, fallopian tubes, and ovaries were delivered through the vagina. The vaginal cuff was sewed close with interrupted figure of eight sutures using 0 Vicryl suture (Johnson & Johnson, New Brunswick, New Jersey, United States (Figure 13).

Step 14. Conclusion of Surgery

**Figure 14:**
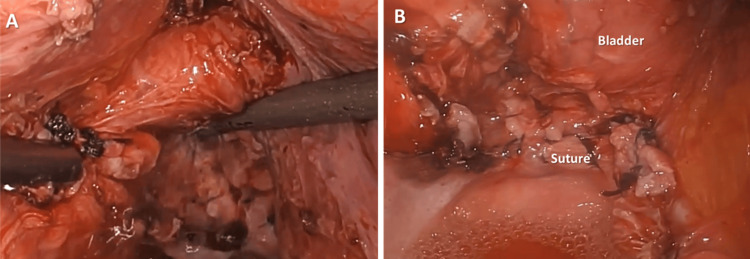
(A) Re-examination of the vaginal cuff and bladder to ensure hemostasis; (B) Flushed and sutured operative field.

The pelvis was irrigated and cleared of all clots. The vaginal cuff and bladder were examined. Bleeding capillaries were cauterized until hemostasis was achieved (Figure 14).

## Discussion

LAVH appears to be the optimal approach in uterus removal, especially in patients with adhesions and previous cervical procedures. This approach takes advantage of direct laparoscopic observation and the less traumatic vaginal surgery.

As a result of previous surgeries, the patient in the current report had significant abdominal and pelvic adhesions. Operative findings were significant for omental adhesions along the width of the anterior abdominal wall, adhesions of the colon to the left side wall, and dense adhesion of the bladder to the lower uterine segment. The cervix was largely absent due to the prior LEEP and cone procedure resulting in a truncated distance between the bladder and uterus, which was further compounded by dense adhesions of the bladder to the lower uterine segment resulting in an ill-defined demarcation of the anterior cul-de-sac. Owing to the prior cone procedure technique of placing stay sutures on each lateral margin of the cervix to decrease bleeding, the anatomical distance between the uterine vessels and the ureter was also reduced.

These alterations of the normal anatomical planes significantly increased the risk of bladder and ureteral injury. Therefore, care was taken to trace and identify the ureters as they enter the pelvic brim and course through the pelvis and into the bladder tunnel. Care was employed to partition the ureter away from the ligation points of the IP ligament containing the ovarian vessels and the cardinal ligaments containing the uterine vessels. Knowledge of the avascular spaces of the pelvis is vital to avoid excess bleeding and to ensure clear visualization of structures [12].

While partitioning the colon from the left fallopian tube and broad ligament to identify the ureter, the posterior peritoneum was cauterized and cut to enter Latsko’s space in the avascular pararectal plane. The ureter was then traced as it traversed below the uterine vessels in the cardinal ligament by dissecting the avascular lateral paravesical space bilaterally and extending this dissection to the avascular medial paravesical space. The avascular vesicovaginal space was then entered and developed further dissecting the bladder away from the lower uterine segment. 

Certain patient factors, however, may preclude a vaginal approach, such as patients with atrophic or absent cervix secondary to cervical procedures such as conization, nulliparity which limits vaginal access and accommodation, severe adhesive disease secondary to endometriosis which may preclude a safe blind entry into the cul-de-sac, and enlarged fibroid uteri.

## Conclusions

We provide a safe and minimally invasive method for hysterectomy with detailed explanations of our procedure. LAVH combines the benefits of two separate approaches: laparoscopic and vaginal. It also helps to avoid the insufficiencies of these methods. However, the laparoscopically-assisted vaginal approach may be challenging, especially in women with concomitant conditions such as adhesions and cervical absence, which are frequently present.
